# Synthesis and Antileishmanial Activity of Lipophilic Aromatic Aminoalcohols

**DOI:** 10.1100/tsw.2010.106

**Published:** 2010-06-14

**Authors:** Roberta Novaes Reis Corrales, Liliane Sena Pinheiro, Elaine Soares Coimbra, Adilson David Da Silva, Mireille Le Hyaric

**Affiliations:** ^1^ Departamento de Química, Universidade Federal de Juiz de Fora Campus Universitário, Juiz de Fora, Minas Gerais, Brazil; ^2^ Departamento de Parasitologia, Microbiologia e Imunologia Universidade Federal de Juiz de Fora Campus Universitário, Juiz de Fora, Minas Gerais, Brazil

**Keywords:** aminoalcohols, antileishmanial activity, Leishmania, polyamines

## Abstract

In this work, we report on the preparation and evaluation of the *in vitro* antileishmanial activity of a series of lipophilic aromatic aminoalcohols. All compounds were assessed for their *in vitro* activity against promastigotes of three *Leishmania species* The most lipophilic aminoalcohols bearing an aliphatic moiety with eight to 12 carbon atoms displayed a good activity against *L. amazonensis* and *L. major*, and two of them also showed antiproliferative activity against *L. chagasi*. The best results were obtained for the N-dodecanoyl ethylenediamine derivative and for N-decyl aminoalcohol (IC_50_ = 5.2 and 0.7 *μ*M, respectively).

## INTRODUCTION

Leishmaniasis is an infective parasitic disease, endemic in the American, African, and Asian tropical countries, with an estimated 1.5–2 million cases per year in 88 countries, and it is classified as one of the "most neglected diseases"[1]. Leishmaniasis is a major vector-borne disease caused by more than 20 species of the protozoan genus *Leishmania*[2]. Human leishmanial infection can manifest itself in three most common forms: cutaneous leishmaniasis, mucocutaneous leishmaniasis, or visceral leishmaniasis (commonly known as Kala-azar). This last clinical manifestation produces severe weight loss, anemia, and systemic impairment, leading to death when untreated. The fatality rate is almost 100% in children and women from poor families[3,4].

Drugs currently in use, such as the antimony derivative glucantime, bis-amidines, pentamidine, and the glycomacrolide amphotericin B, display high liver and heart toxicities and lead to clinical resistance after a few weeks of treatment[5,6]. Other limitations to the use of these drugs include the cost of the treatment and the need for parenteral administration[5]. All these factors contribute to compliance difficulties and treatment failures, and there is a real need to discover new potent and selective agents, either natural or synthetic, for the treatment of this increasing parasitosis.

*Leishmania* and other protozoa belonging to the trypanosomatid family have distinct polyamine metabolisms compared to mammalian cells, opening the possibility of identifying new targets for antiparasitic drug development[7,8,9,10,11]. Polyamines such as putrescine and spermidine are aliphatic organic compounds that play vital roles in several eukaryotic cellular processes, such as growth, protein/nucleic acid synthesis, and macromolecular biosynthesis[12,13]. Trypanosomes are unusual organisms in that spermidine is also used in the synthesis of trypanothione, which is both unique and essential to those parasites. The cloning, expression, purification, and crystallization of trypanothione reductase in *Leishmania* species was recently reported[14,15].

We describe in the present paper the synthesis of lipophilic aromatic aminoalcohols and their *in vitro* biological activities against *Leishmania* promastigote forms. These types of compounds, bearing a covalent bonded aliphatic chain attached to an aminoalcohol fragment, could interact with membrane lipids and be transported into the cytoplasm where they can possibly interfere with the lipid or polyamine transport or metabolism of the parasite[16].

## MATERIALS AND METHODS

### Chemicals

The aminoalcohol derivatives **3a-d** were prepared in 63–80% yield by treatment of 2-phenyl-ethylbromide **1** with aminoalcohols **2a-d,** as previously described[17,18,19,20] (Scheme 1). The phenethyl glycidyl ether **4**, prepared using a simple synthetic method[20], was treated in ethanol at 70ºC for 24 h with the aminoalcohols **2a-d** (Scheme 2), the amides **6a-c**[21], and with the amines **8a,b**, leading, respectively, to aminoalcohols **5a-d**, **7a-c,** and **9a,b** in 56–84% yield (Scheme 3). All the compounds were characterized by NMR and IR spectroscopy[22].

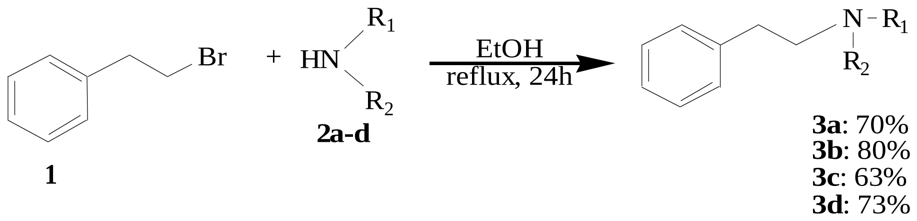

**a-** R=H; $R_1$= $(CH_2)_2OH$; **b-** R=H; $R_1$= $(CH_2)_3OH$; **c-** R=H; $R_1$= $C(CH_3)_2CH_2OH$; **d-** R=$R_1$= $CH_2CH_2OH$

**SCHEME 1.** Synthesis of aromatic aminoalcohols **3a-d.**

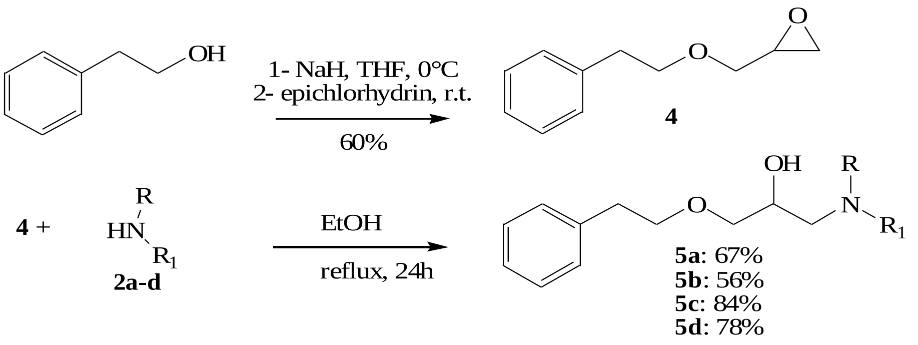

**a**: R=H, $R_1$=$(CH_2)_2OH$; **b**: R=H, $R_1$=$(CH_2)_3OH$; **c**: R=H, $R_1$=$C(CH_3)_2CH_2OH$; **d**: R=$R_1$=$CH_2CH_2OH$

**SCHEME 2**. Synthesis of aromatic aminoalcohols **5a-d** from phenethyl alcohol.

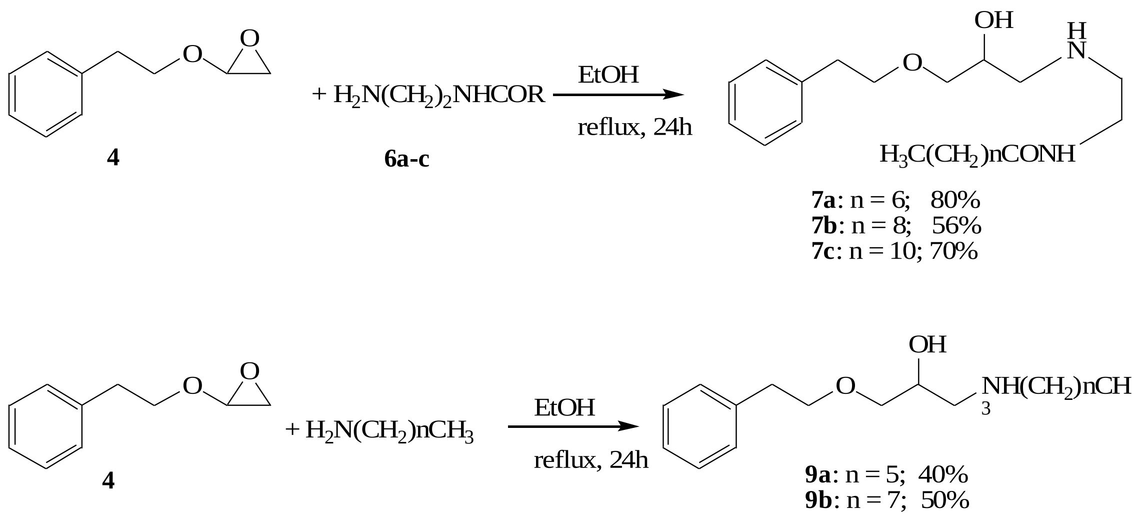

**SCHEME 3.** Synthesis of aminoalcohols **7a-c** and **9a,b**.

## Biological Assay

In this study, promastigotes of *L. chagasi* (MHOM/Br/74/PP75), *L. amazonensis* (IFLA/Br/67/PH8), and *L. major* (MRHO/SU/59/P) were used. The viability of the promastigotes was determined by the colorimetric 3-(4,5-dimethylthiazol-2-yl)-2,5-diphenyl-tetrazolium bromide (MTT) method based on tetrazolium salt reduction by mitochondrial dehydrogenases[23]. The results are expressed as the concentration inhibiting the parasite growth by 50% ($IC_{50}$) after a 3-day incubation period with the tested compound. Amphotericin B (Cristália-São Paulo) was used as the reference standard. $IC_{50}$ values were obtained from at least two independent experiments performed in triplicate, and *GraFit* Version 5 software (Erithacus Software Ltd., Horley, U.K.) was used for the analysis of the data.

## RESULTS AND DISCUSSION

Among the evaluated compounds, the less lipophilic *N*-phenethylethanolamine **3a** and its analogues **3b-d** were inactive against all *Leishmania* species promastigotes at tested concentrations (Table 1). In the aminodiol series **5a-d**, compounds **5a** and **5c** showed a good activity against *L. major* promastigotes ($IC_{50}$ values of 13.6 and 4.6 μM, respectively). Lipophilic aminoalcohols **7a-c**, bearing an aliphatic amide moiety, were active against both *L. amazonensis* and *L. major* promastigote forms. Compound **7b**, a decanoylethylenediamine derivative, showed the best activity against *L. amazonensis* ($IC_{50}$ of 18.6 μM) and *L. major* ($IC_{50}$ of 5.2 μM).

The two alkylated aminoalcohols (**9a,b**) displayed the best inhibitory activity against all *Leishmania* promastigotes tested, with $IC_{50}$ values ranging from 0.7 to 49.0 μM. Compound **9b**, bearing an octyl chain, was the most efficient against the three species with $IC_{50}$ values of 18.5, 0.7, and 30.0 μM, for *L. amazonensis*, *L. major*, and *L. chagasi* promastigote forms, respectively.

From these results, we can observe a pattern of sensitivity of *Leishmania* species to the tested compounds. *L. major* promastigotes were the most sensitive to the tested aminoalcohols, with $IC_{50}$ values <20 μM. This strain also showed the smallest $IC_{50}$ between the 14 compounds tested (0.7 μM). This pattern of sensitivity of the parasites to different drugs is not surprising as it has been reported in several studies[23,24,25,26].

**TABLE 1**
***In vitro*** **50% Inhibitory Activity against Promastigotes of Three** ***Leishmania*** **Species**

| **Compounds** | **IC50 (µM)** | | |
|---|---|---|---|
| | ***L. amazonensis*** | ***L. major*** | ***L. chagasi*** |
| **3a** | >87.0 | >87.0 | >87.0 |
| **3b** | >87.0 | >87.0 | >87.0 |
| **3c** | >87.0 | >87.0 | >87.0 |
| **3d** | >87.0 | >87.0 | >87.0 |
| **5a** | >87.0 | 13.6 ± 2.2 | >87.0 |
| **5b** | >87.0 | >87.0 | >87.0 |
| **5c** | >87.0 | 4.6 ± 5.3 | >87.0 |
| **5d** | >87.0 | >87.0 | >87.0 |
| **7a** | 49.0 ± 0.7 | 14.4 ± 1.4 | N.T.* |
| **7b** | 18.6 ± 4.6 | 5.2 ± 0.3 | >87.0 |
| **7c** | 46.4 ± 2.0 | 19.7 ±2.9 | >87.0 |
| **9a** | 37.3 ± 3.0 | 11.4 ± 0.2 | 27.5 ± 0.3 |
| **9b** | 18.5 ± 2.9 | 0.72 | 30.0 ± 0.2 |
| AmB** | 0.4 | 0.32 | 1.9 ± 0.2 |

* N.T., Not tested.

** AmB, Amphotericin B (control drug).

The results point to the importance of lipophilicity for antileishmanial activity: the two most active compounds are alkylated (**9b**) or acylated (**7b**) aminoalcohols, while none of the less lipophilic compounds (**3a-d**) was active.

## CONCLUSION

This work describes the preparation and biological evaluation of several aminoalcohols prepared by the reaction of aromatic halides and aromatic glycidyl ethers with aminoalcohols (**3a-d** and **5a-d**), aliphatic amides (**7a-c**), and aliphatic amines (**9a,b**). Five compounds (**7a-c** and **9a,b**) displayed a good activity against *L. amazonensis*, seven compounds (**5a**, **5c**, **7a-c,** and **9a,b**) were active against *L. major*, and only two (**9a,b**) were active against *L. chagasi*. These results show that the lipophilicity of the respective aminoalcohols is of great importance for antileishmanial activity, as compounds **7b** and **9b**, containing aliphatic side chains with 10 or eight carbons, showed the best activity against *L. amazonensis* and *L. major* promastigote forms.

## ACKNOWLEDGMENTS

The authors gratefully acknowledge UFJF, CAPES, and CNPq for fellowships. This research was supported by FAPEMIG.
